# Preconditioning with far-infrared irradiation enhances proliferation, cell survival, and migration of rat bone marrow-derived stem cells via CXCR4-ERK pathways

**DOI:** 10.1038/s41598-017-14219-w

**Published:** 2017-10-20

**Authors:** Yun-Mi Jeong, Xian Wu Cheng, Sora Lee, Kyung Hye Lee, Haneul Cho, Jung Hee Kang, Weon Kim

**Affiliations:** 1 Division of Cardiology, Department of Internal Medicine, Kyung Hee University Hospital, Kyung Hee University, Seoul, Republic of Korea; 2 The Department of Cardiology, Yanbian University Hospital, Yanji, China

## Abstract

Far-infrared radiation (FIR) has been shown to exert positive effects on the cardiovascular system. However, the biological effects of FIR on bone marrow-derived stem cells (BMSCs) are not understood. In the present study, BMSCs were isolated from rat femur bone marrow and cultured *in vitro*. To investigate the effects of an FIR generator with an energy flux of 0.13 mW/cm^2^ on rat BMSCs, survival of BMSCs was measured by crystal violet staining, and cell proliferation was additionally measured using Ez-Cytox cell viability, EdU, and Brd U assays. FIR preconditioning was found to significantly increase BMSC proliferation and survival against H_2_O_2_. The scratch and transwell migration assays showed that FIR preconditioning resulted in an increase in BMSC migration. qRT-PCR and Western blot analyses demonstrated that FIR upregulated Nanog, Sox2, c-Kit, Nkx2.5, and CXCR4 at both the mRNA and protein levels. Consistent with these observations, PD98059 (an ERK inhibitor) and AMD3100 (a CXCR4 inhibitor) prevented the activation of CXCR4/ERK and blocked the cell proliferation and migration induced by FIR. Overall, these findings provide the first evidence that FIR confers a real and significant benefit on the preconditioning of BMSCs, and might lead to novel strategies for improving BMSC therapy for cardiac ischemia.

## Introduction

The best clinical therapy for severe ischemic heart disease (IHD) has been heart transplantation^1^. Due to limited heart donors, immune rejection, and infection, transplantation is not always clinically feasible^1^. Recently, many patients with IHD have participated in bone marrow cell-derived stem cell (BMSC)-based therapy^1–4^. Cumulative successful results from preclinical and early phase clinical trials have indicated that BMSC therapy could revolutionize the surgical treatments of patients with IHD^1–4^. Although we have great interest in the results of additional ongoing clinical trials involving BMSCs, many research groups around the world are now moving towards enhancing the relatively low therapeutic efficacy of BMSC-based therapy.

A critical issue in bench-to-bedside studies of BMSC-based therapy that remains is how to improve the safety and efficacy of such therapies^1,2^. Because different microenvironments exist during cell propagation *in vitro* or in injured tissue after injection, BMSCs need to be able to protect themselves from the potentially detrimental effects of thermal shock, food shortage, oxidative stress, and ischemia^5^. Thus it is important to improve the survival and therapeutic effects of BMSCs before BMSC-based therapies are used for patients with severe IHD. Recently, MSC preconditioning has emerged as an attractive therapeutic strategy against the harsh ischemic microenvironment of the heart^6–8^. Several studies have demonstrated that exposure to hypoxia, anoxia, acidosis, heat shock, cytokines, low-level laser radiation (630 nm, 850 nm), or pharmacological treatments prior to cell injection into the damaged tissue help progenitor cells withstand the harsh ischemic microenvironment of the heart^6–8^.

Far-infrared radiation (FIR) is a subdivision of the electromagnetic spectrum in the wavelength range of 5.6 – 1000 μm^9^. The specific effects of FIR on BMSC preconditioning remain unknown. Previous studies have reported that FIR treatment produces both thermal and non-thermal effects, including increased artery blood flow and peripheral blood circulation, improved endothelial function, alleviation of fatigue and pain, reduced blood pressure, and promotion of capillary dilation^9,10^. In this study, we explored a strategy to improve BMSC preconditioning by FIR and tested the potential of FIR preconditioning to enhance proliferation, cell survival, and migration of BMSCs.

## Results

### FIR^50 min^ preconditioning improves proliferation, cell survival, and migration of BMSCs

**Figure 1:**
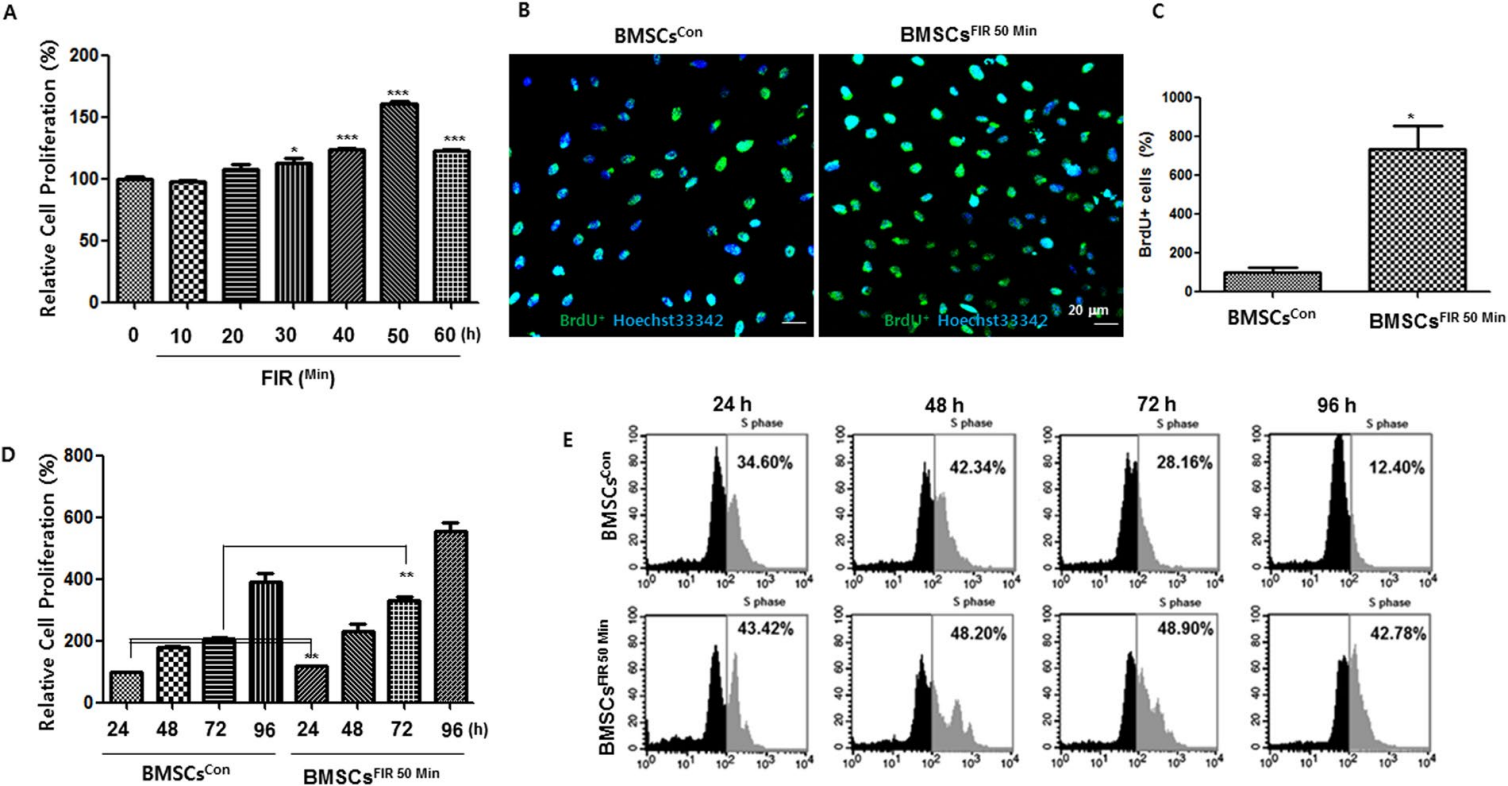
FIR promotes BMSC proliferation in a dose-and time-dependent manner. (**A**) The results of the cell proliferation assay used to verify the preconditioning effects of FIR on BMSCs. After FIR treatment at the indicated duration time, the cells were incubated for 72 h and were then analyzed by the EZ-Cytox assay. (**B**) BrdU was incorporated in both the BMSCs^con^ and BMSCs^FIR 50 min^ for 72 h. Confocal fluorescence microscopy images of BMSC division and proliferation after BrdU assays were taken, as described in the Materials and Methods section. The incorporated BrdU was stained with anti-BrdU AlexaFluor® 488 monoclonal antibody (green) with cell nuclei counter-stained with Hoechst33342 (blue). Scale bars, 20 μm. (**C**) The percentages of BrdU^+^ cells are shown in the bar graph. (**D** and **E**). At the indicated time points after FIR^50 min^ preconditioning, cell proliferation was measured by the EZ-Cytox assay and the Click-iT® Plus Edu Alexa Fluor ® 488 Flow Cytometry Assay kit, as described in the Materials and Methods. All data represent the mean ± SD of triplicate assays expressed as percentages of the BMSCs^con^. *P < 0.05, **P < 0.01, ***P < 0.001 versus BMSCs^con^.

To determine whether FIR affects BMSC proliferation, rat BMSCs were treated with FIR for a duration of 0, 10, 20, 30, 40, 50, or 60 min. After three days, cell proliferation was assessed with an EZ-Cyto assay and cell counter method. As shown in Figs 1A and S2A, FIR significantly stimulated BMSC proliferation after a duration of 30, 40, 50, or 60 min, and did not affect cell viability (Fig. S3). At 50 min treatment, FIR-treated BMSC proliferation exhibited a 1.5-fold increase. To further quantify the impact of FIR 50 min-treatment on BMSC proliferation, an anti-Brd U fluorescence assay was performed. Consistent with the findings shown in Fig. 1A, confocal images of the BrdU incorporation assay confirmed a larger number of BrdU^+^ for the FIR treated cells compared to the control (BMSCs^con^)(Fig. 1B and C). Furthermore, there were significant and time-dependent differences in growth rate, cell viability, and cell proliferation rate between BMSCs^con^ and BMSCs^FIR 50 min^ (Figs 1D and S2B). At all time points, the BMSCs^FIR 50 min^ demonstrated a significant dose-responsive increase in cell number compared to EdU-positive BMSCs^con^ (1D). Therefore, a duration of FIR 50 min (FIR^50 min^) was chosen as optimal for the preconditioning of BMSCs *in vitro*.

**Figure 2:**
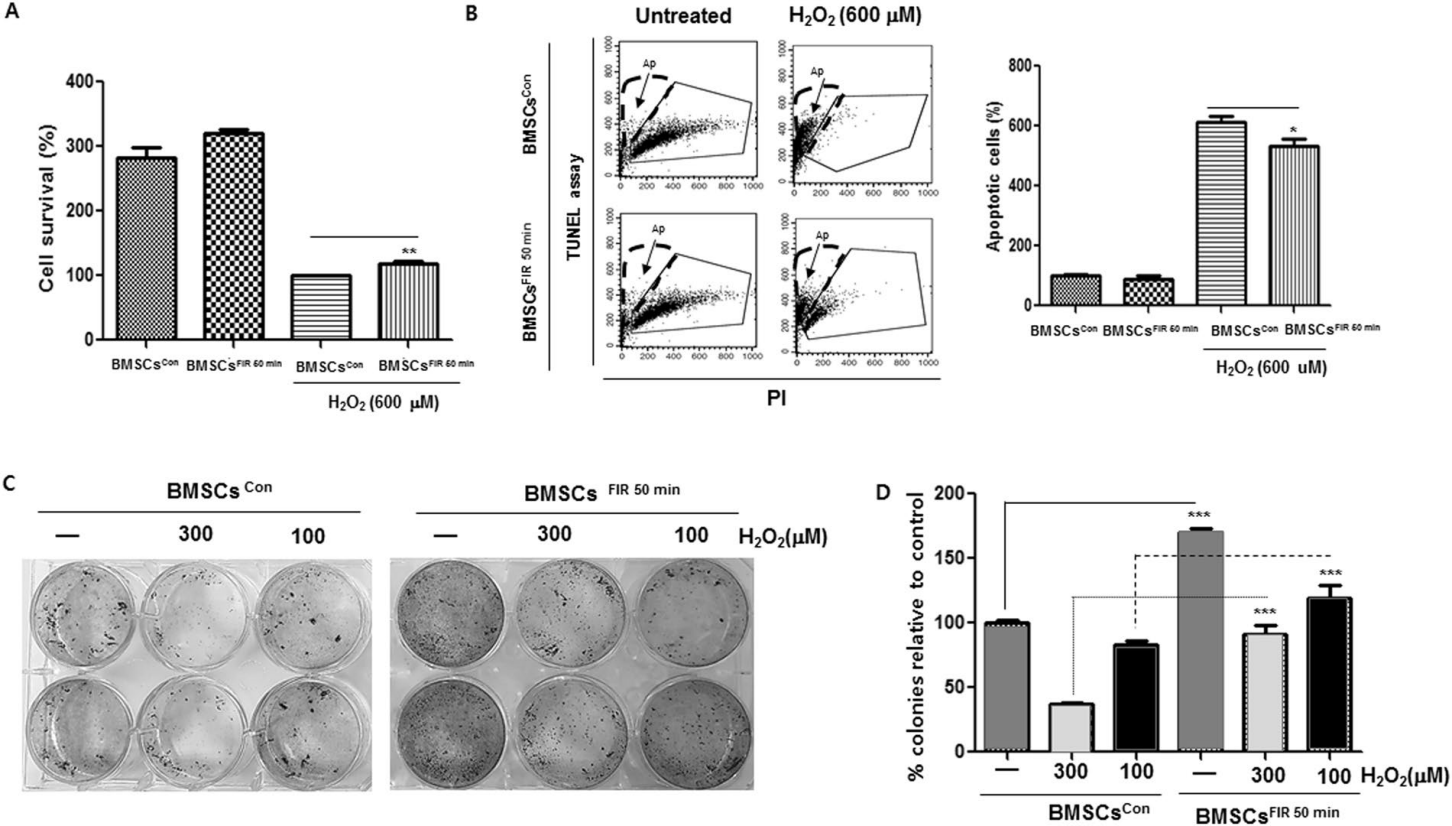
FIR preconditioning increases BMSC survival against H_2_O_2_-induced apoptosis. (**A**) After FIR^50 min^ preconditioning, these cells were treated with H_2_O_2_ (600 μM) for 24 h. The cell viability was quantified using crystal violet staining. (**B**) The apoptotic cells (Ap) were analyzed by flow cytometry using a TUNEL assay, as described in the Materials and Methods. The percentage of Ap was calculated from fluorescence dot plots (bounded within the black broken line). The surviving cells are contoured by a solid gray line. (**C**) The BMSCs^con^ and BMSCs^FIR 50 min^ were treated with H_2_O_2_ (300 μM and 100 μM) for 24 h, transferred to a fresh medium, and incubated for 72 h. Cell proliferation was measured using crystal violet staining. (**D**) Graph of the recovery of proliferative capacity of cells after H_2_O_2_ treatment (300 μM and 100 μM). Data are presented as means ± SD of three independent experiments. ***P < 0.001 vs. the H_2_O_2_-treated BMSCs^con^.

**Figure 3:**
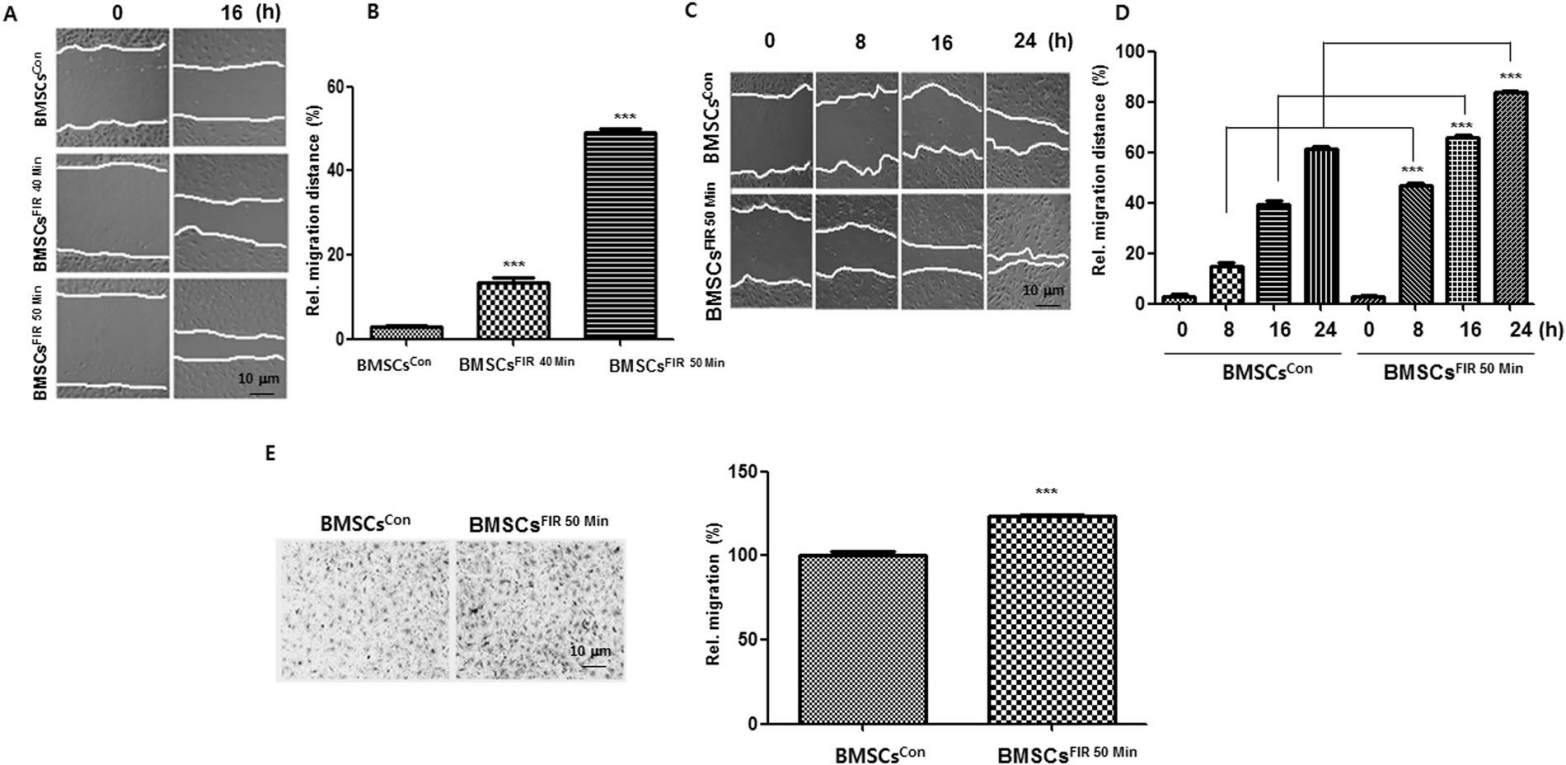
FIR preconditioning accelerates BMSC migration in a dose- and time-dependent manner. (**A**) Phase contrast images of wound healing migration assays in a dose-dependent manner. Wounds were made then treated with FIR, as described in the Materials and Methods section. (**B**) The rate of cell migration in a dose-dependent manner. (**C**) Phase-contrast images for wound healing migration assay over time. Scale bars, 10 μm. (**D**) The rate of cell migration over time. Data represent the mean ± SD of 10 randomly chosen fields expressed as percentages of the BMSCs^con^. ***P < 0.001 compared to the BMSCs^con^. Average rates of wound closure were calculated from three independent experiments. (**E**) Transwell migration assay for BMSCs^con^ and BMSCs^FIR 50 min^ using crystal violet staining.

To examine whether FIR-preconditioning protects BMSCs from oxidative injury, H_2_O_2_ was applied to induce BMSC apoptosis. BMSCs^FIR 50 min^ were better protected against H_2_O_2_ than BMSCs^con^ (Fig. 2A). The TUNEL assays showed that FIR^50 min^ alleviated H_2_O_2_-induced apoptosis (Fig. 2B). We next hypothesized that, if FIR preconditioning were involved in the protection of BMSCs against H_2_O_2_, it would also affect the recovery of proliferative capacity and survival of BMSCs. To test this hypothesis, BMSCs^FIR 50 min^ were treated with H_2_O_2_. After 24 h, the medium was exchanged for fresh medium and incubated for 72 h. The recovery of proliferative capacity of BMSCs^FIR 50 min^ was higher than for BMSCs^con^ (Fig. 2C and D). We next evaluated whether FIR^50 min^ preconditioning affects BMSC migration using the scratch migration and transwell migration assays. Representative photographs of the scratch migration and transwell migration assays are shown in Fig. 3. The migration rate of the BMSCs^FIR 50 min^ was significantly higher than for the BMSCs^con^.

### FIR^50 min^ preconditioning upregulates cardiac-specific and pluripotency-associated markers

**Figure 4:**
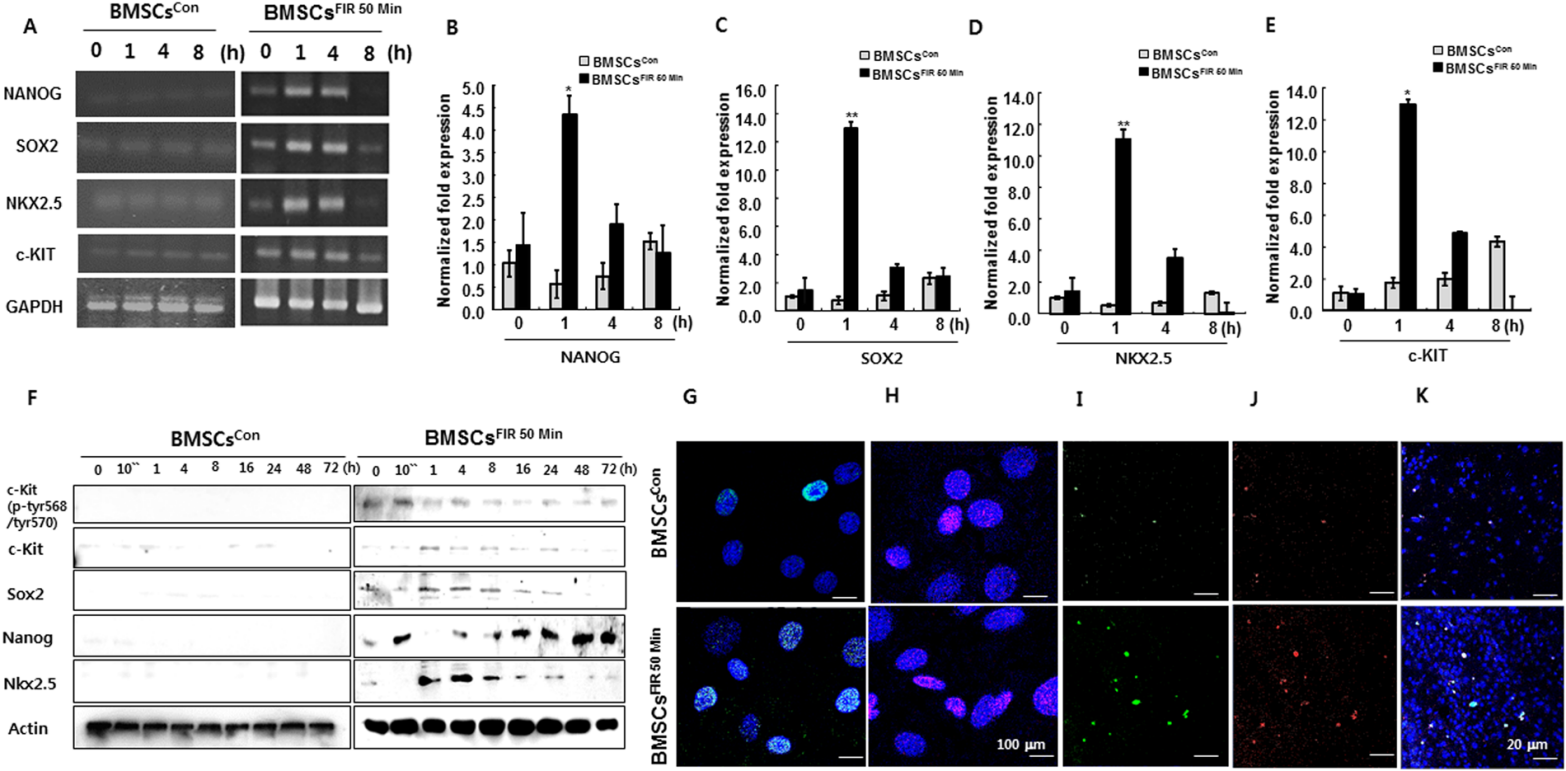
Higher expression of pluripotency markers and cardiac lineage-associated markers in BMSCs^FIR 50 min^. (**A**) Representative agarose gel images of RT-PCR products for targeted genes in BMSCs^con^ and BMSCs^FIR 50 min^ at 0, 1, 4, and 8 h. (**B**–**E**) qRT-PCR analysis of relative targeted gene expression in BMSCs^con^ (gray) and BMSCs^FIR 50 min^ (black). (**F**) Western blot analysis of targeted proteins. (**G**–**J**) Confocal images of Sox2^+^ (Cyan, 100 μm), Nanog^+^ (Pink, 100 μm), c-Kit^+^ (green, 20 μm), and Nkx2.5^+^ (red, 20 μm)-expressing cells. (**K**) Overlay of c-Kit^+^ and Nkx2.5^+^ -expressing cells. Data were analyzed using AVOVA followed by Tukey’s *post hoc tests* and displayed as mean ± SD (n = 6). *P < 0.05 and **P < 0.01 versus corresponding controls.

**Figure 5:**
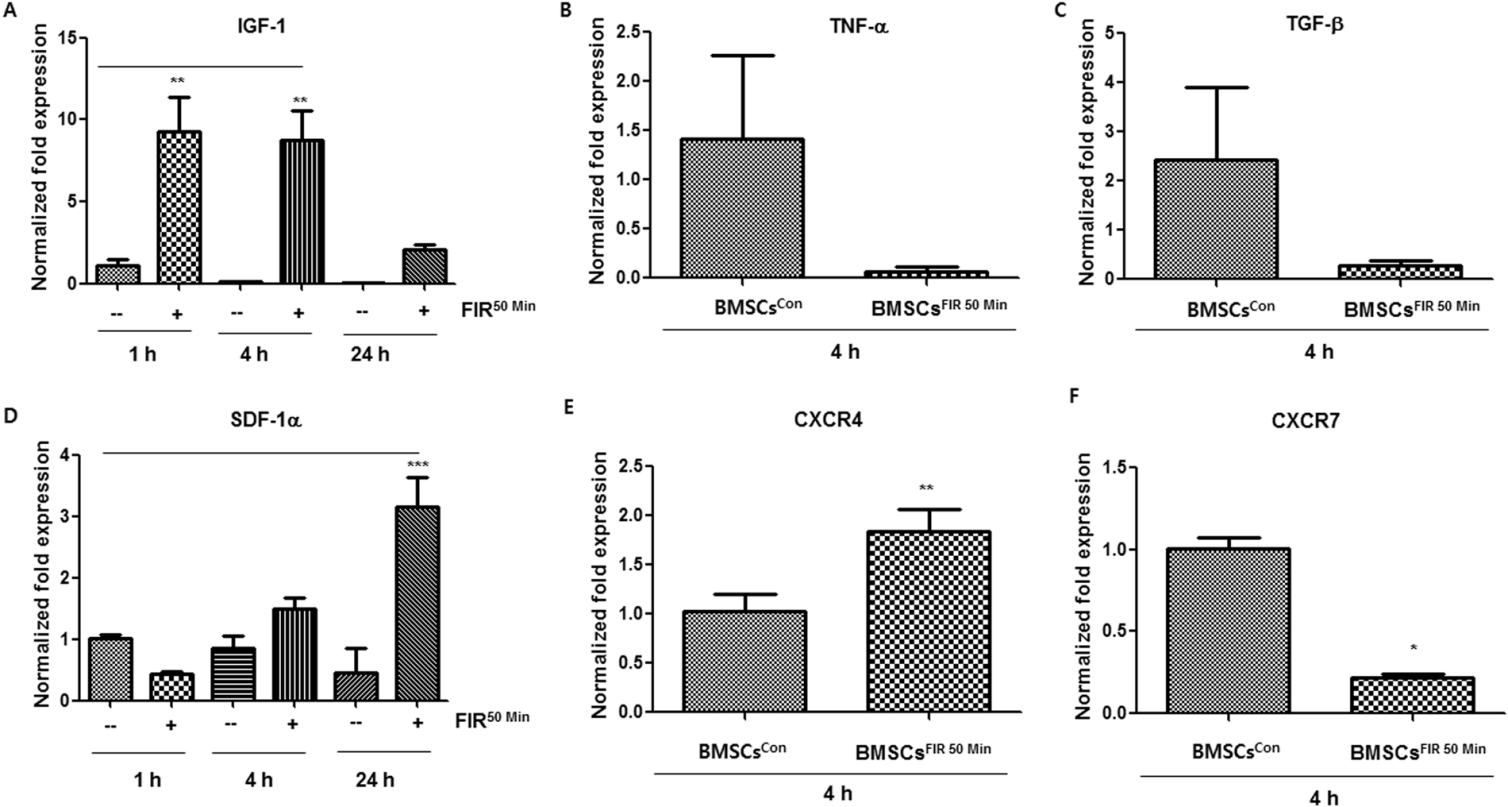
BMSCs^FIR 50 min^ overexpress SDF-1α and IGF-1 at the mRNA level. (**A**–**F**) Graphs of qRT-PCR analysis results depicting the mRNA expression of IGF-1, TNF-α, TGF-β, SDF-1α, CXCR4, and CXCR7 in BMSCs^con^ (−) and BMSCs^FIR 50 min^ ( + ) at the indicated times after FIR^50 min^ preconditioning. Data were analyzed using Student’s t-test or AVOVA followed by Tukey’s *post hoc tests* and displayed as mean ± SD (n = 6). *P < 0.05, **P < 0.01, and **P < 0.0001 versus corresponding controls.

To further verify the preconditioning effects of FIR on BMSCs, we carefully selected 19 well-studied major cardiac-specific and pluripotency-associated markers (Table S1). Using qRT-PCR analysis, we found that mRNA levels of NANOG, SOX2, c-KIT, and NKX2.5 significantly increased in a time-dependent manner in BMSCs^FIR 50 min^ (Fig. 4A–E). These observations were confirmed by Western blot analysis and immunofluorescence staining, which showed elevated expression of Nanog, Sox2, c-Kit, and Nkx2.5 in BMSCs^FIR 50 min^ during various time frames (Fig. 4F–K). If FIR preconditioning were involved in the upregulation of pluripotency-associated and cardiac-specific markers, it may also affect the expression of paracrine factors in BMSCs. To address this possibility, the mRNA levels of paracrine factors in BMSCs^con^ and BMSCs^FIR 50 min^ were measured using qRT-PCR analysis. We detected high levels of IGF-1 and SDF-1α mRNA in the BMSCs^FIR 50 min^, whereas mRNA for TNF-α and TGF-β were lower in BMSCs^FIR 50 min^ than in BMSCs^con^ (Fig. 5A–D). Interestingly, BMSCs^FIR 50 min^ also expressed a high level of CXCR4 mRNA and downregulated expression of CXCR7 (Fig. 5E and F).

### CXCR4/ERK activation is essential to FIR-mediated BMSC preconditioning

**Figure 6:**
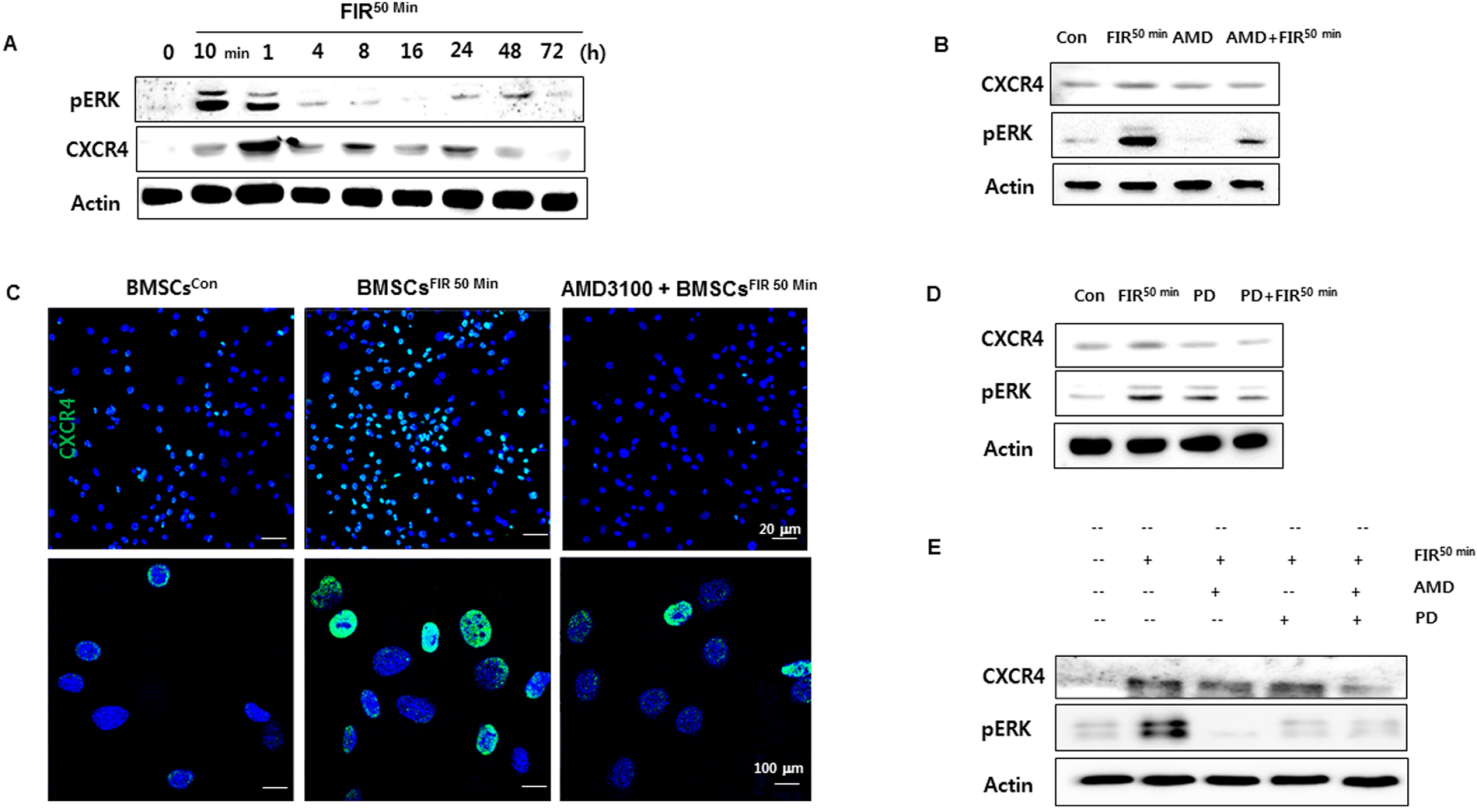
CXCR4/ERK activation is crucial for the FIR-preconditioned BMSCs. (**A**) Western blot analysis showing the activation of CXCR4 and ERK in BMSCs^FIR 50 min^ over time. (**B** and **C**) The effects of AMD3100 (10 μM) on BMSCs^con^ and BMSCs^FIR 50 min^ –mediated upregulation of CXCR4 were detected by Western blot analysis and confocal fluorescence microscopy with indicated antibodies. Scale bars are 20 μm (upper) and 100 μm (lower). (**D** and **E**) The effect of FIR on CXCR4/ERK-mediated BMSCs pretreated with AMD3100 and/or PD98059. These cells were pretreated with one or both of these inhibitors (10 μM) for 2 h, then FIR^50 min^ preconditioning was performed, and all BMSCs incubated for 1 h.

**Figure 7:**
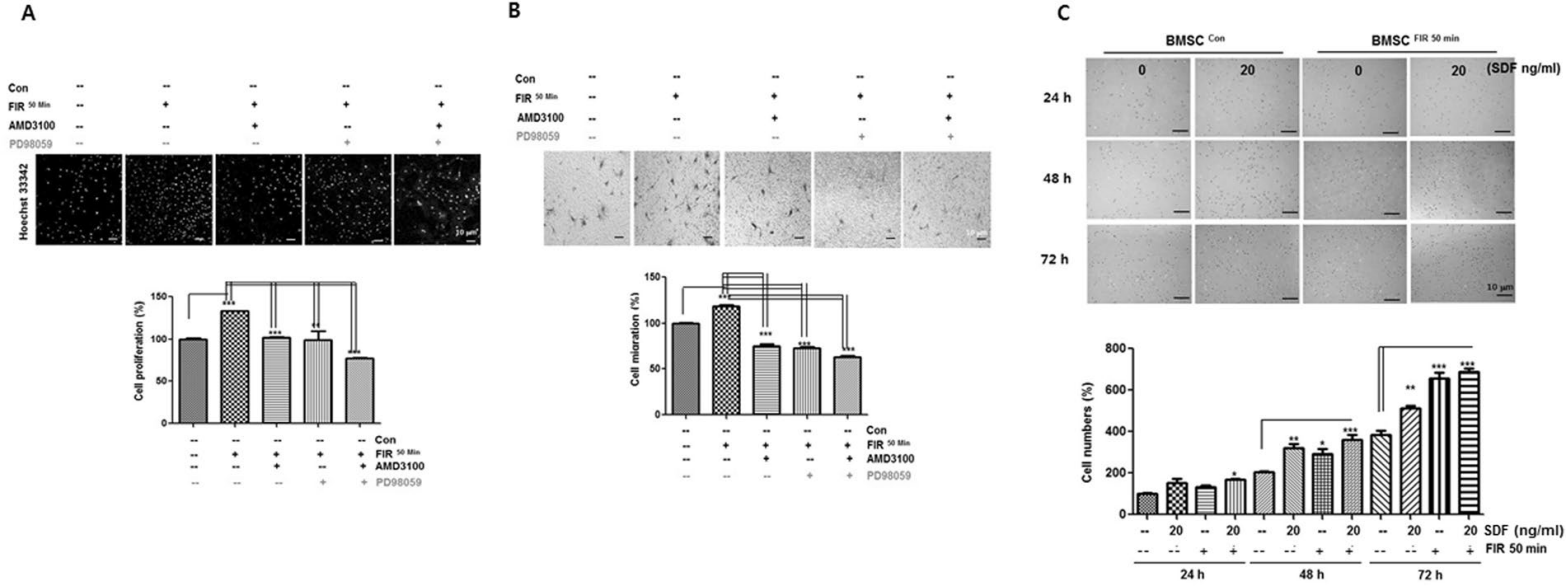
The effects of AMD3100, PD98059, and SDF-1α on FIR-preconditioned BMSC proliferation and migration. (**A**) Confocal images of Hoechst33342-stained BMSCs^con^ and BMSCs^FIR 50 min^ pre-treated with one or both of these inhibitors. These cells were pretreated for 2 h, then the FIR 50 min was performed, and all cells incubated for 72 h. Scale bars are 10 μm. Graph indicating the proliferation of these cells with one or both of these inhibitors, as identified using an EZ-Cytox assay. (**B**) Phase contrast images of transwell migration assay for BMSCs^con^ and BMSCs^FIR 50 min^ with one or both of these inhibitors. Graph indicating the rate of cell migration. (**C**) Time lapse images of BMSCs^con^ and BMSCs^FIR 50 min^ with and without pretreatment of SDF-1α (20 ng/ml). Graph of proliferation rates, using a cell counter at the indicated time points for representative samples, shown as percentage increases compared to BMSCs^con^ at t = 24 h. All data represent the mean ± SD of three independent experiments in triplicate assays expressed as percentages of the BMSCs^con^. For all pretreatments *P < 0.05, **P < 0.01, ***P < 0.001 versus BMSCs^con^. (−, untreated; +, FIR^50 min^ preconditioning).

To determine how FIR affects BMSC preconditioning via CXCR4 expression, Western blot analyses were performed to detect the activation of CXCR4 and phosphorylation of ERK, which are both known to play a role in regulating cell proliferation and migration-related signaling pathways. The phosphorylation of ERK was markedly activated 10 min after FIR^50 min^ preconditioning (Fig. 6A). Moreover, FIR^50 min^ preconditioning increased the level of CXCR4 protein at 1 h (Fig. 6A). We next pretreated the control and FIR treated cells with the specific inhibitors AMD3100 (known as an antagonist of CXCR4 binding to SDF-1) or PD98059 (known as a synthetic ERK1/2 inhibitor) or both, to further verify the upregulation of CXCR4 and ERK by FIR^50 min^ preconditioning. The results show that these inhibitors prevented the upregulation of CXCR4 and ERK activation in BMSCs^FIR 50 min^ (Fig. 6B–D), as well as prevented the enhancement of cell proliferation and migration induced by FIR^50 min^ preconditioning (Fig. 7A and B). Likewise, FIR^50 min^ preconditioning facilitated SDF-1α-induced BMSC migration, and this effect was diminished by the monotherapy and combination therapy of the inhibitors (Fig. 7B). Moreover, we observed that exogenous SDF-1α significantly stimulated cell proliferation in BMSCs^con^ group whereas it exhibited a minor effect in the BMSCs^FIR50 min^ group (Fig. 7C). These findings thus indicate that FIR^50 min^ preconditioning appears to modulate BMSC cellular functions through the activation of the SDF-1α/CXCR4-ERK1/2 signaling pathway.

## Discussion

Our findings highlight the novel insight that FIR preconditioning significantly promotes BMSC proliferation, migration, cell survival, and recovery against H_2_O_2_. In general, FIR radiation transfers energy to the human body and manifests a wide variety of biological effects, including improvement of ischemic lesions^9,10^. Although the biological activities of FIR in the preconditioning of BMSCs by *in vitro* manipulation are not fully understood, our findings corroborate previous studies of wavelength in the red range^11–17^. For example, red (630 nm) and near infrared (850 nm) light-emitting diodes enhance the migration of MSCs derived from rat bone marrow^11–13^. Other studies have demonstrated that a diode (Ga-As) laser of wavelength 804 nm promots the proliferation of BMSCs and cardiac stem cells^14–16^. Interestingly, preconditioning using this wavelength has exhibited a cardioprotective effect on myocardial ischemia/reperfusion (I/R) injury *in vivo* ^16^. Another study has suggested that postconditioning with FIR increases heme oxygenase-1 expression and protects against I/R injury in rat testes^17^.

The present study shows for the first time that the preconditioning effects of FIR on BMSCs include the activation of CXCR4 and ERK. We also show that FIR strongly induced the expression of SDF-1α and IGF-1 at the mRNA level, while downregulating TNF-α and TGF-β. AMD3100 and PD89059 treatment further confirmed that the preconditioning ability of FIR occurs via activation of CXCR4 and ERK. CXCR4 overexpression is a key part of enhancing the efficacy of stem cell homing and stem cell preconditioning through paracrine signaling mechanisms^18–23^. CXCR4 overexpression in human adipose tissue-derived stem cells has been demonstrated to improve homing and engraftment in an animal limb ischemia model^21^. In the case of acute kidney injury, the overexpression of CXCR4 has been shown to enhance the repair ability of BMSCs, increasing the homing of BMSCs and increasing the release of cytokines^22^. In a case of skin injury repair, CXCR4 overexpression in BMSCs promoted wound healing in a SDF-1-expression-dependent manner^23^.

One of the limitations of the present study is that it was challenging to separate the effect of FIR-mediated preconditioning on BMSC migration and proliferation. Nevertheless, we have clearly demonstrated by the colony and transwell migration assays that FIR-mediated preconditioning facilitates BMSC migration and proliferation. Additionally, we could not determine whether FIR-mediated preconditioning promotes rat BMSC differentiation. The present study also does not provide evidence as to whether FIR-preconditioned BMSCs can preferentially migrate to damaged cardiac tissue. Several reports have suggested that CXCR4-enhanced BMSC preconditioning may beneficially effect the mobilization or transplantation of cells into damaged areas in rats^24–26^. For example, preconditioning with tetramethylpyrazine (TMP) significantly upregulated the protein levels of SDF-1 and CXCR4 in BMSCs^24^. In addition, transplantation of BMSCs preconditioned with TMP demonstrated more improved functional outcomes compared to controls in rat ischemic stroke models^24^.

Other papers have reported that dimethyloxalylglycine (DMOG), which is a hypoxia inducible factor hydroxylase inhibitor, stimulates the early upregulation of myocardial CXCR4 expression, which in turn induces cardiac improvement in acute myocardial infarction^25,26^. Of note, DMOG-preconditioned BMSCs reduced heart infarct size and promoted heart function^26^. Consistent with these previous studies, FIR preconditioning should be further investigated in order to generate new BMSC-based photobiomodulation therapies. In conclusion, the present study clearly indicates the ability of FIR to promote BMSC proliferation, migration, cell survival, and cell recovery. Therefore, our novel findings might have practical application in the field of BMSC therapy, offering a simple, easy, and non-invasive enhancing strategy for improving the survival and engraftment rates of BMSCs transplanted into the infarcted heart.

## Materials and Methods

### Isolation and culture of rat BMSCs

The present study was reviewed and approved by the Institutional Ethics Committee on Animal Resources of Kyung Hee University Hospital (licensing ID KHMC-IACUC:2015-028), and it conformed to the guiding principles of the ‘Guide for the Care and Use of Laboratory Animals.’ BMSCs were isolated and harvested from 6- week-old male Sprague-Dawley rats (n = 20) as described previously^16^. In brief, BMSCs were acquired by flushing the cavities of femurs and tibias with a basal MSCGM hMSC medium with 10% FBS. Collected BMSCs were seeded onto dishes with an MSCGM bulletkit medium with a MSCGM hMSC SingleQuot Kit. The cells were cultured for 1 week in a complete medium at 37°C in a 5% CO_2_ incubator. The rat BMSCs of the P2 passages were used.

### FIR preconditioning

A WS ^TM^TY101N emitter FIR therapy unit (WS Far Infrared Medical Technology CO, Ltd, Taipei, Taiwan) was used for the preconditioning of the BMSCs. The electrified ceramic plates of this emitter generate electromagnetic waves with wavelengths in the range of 3 – 25 mm (peak, 8.2 mm). The irradiating power density is 10 and 20 mW/cm^2^ when the top radiator is set at a distance of 30 cm above the culture plate surface for the indicated times. To evaluate whether FIR induced the thermal or non-thermal effects on culture plates, we used a thermometer. During the 60 min of FIR treatment, the plate temperatures remained under 30°C. BMSCs exposed to FIR for 50 min (BMSCs^FIR50 min^) were used to ensure the cells were sufficiently “preconditioned”, and non-irradiated BMSCs (BMSCs^con^) were used as a control for all experiments.

### Quantitative real-time transcription polymerase chain reaction (qRT-PCR)

cDNA was synthesized from 3 μg of each sample’s RNA using an AccuPower®RocketScript^TM^ Cycle RT PreMix (dN12). PCR was performed using the AccuPower®ProFi Taq PCR PreMixture. Thermal cycling consisted of an initial denaturation step at 94°C for 5 min, followed by over 40 cycles of denaturation at 94°C for 30 min, annealing at the indicated temperature for 30 s, and extension at 72°C for 10 min. A list of the primers utilized is presented in Table S1. qRT-PCR was carried out following standard procedures using SYBR®Green Mix with primers (Table S1). qRT-PCR reactions were performed in triplicate in the StepOnePlus real-time PCR system (Applied Biosystems). Quantitative measures from all data were obtained using the delta-delta-CT method with normalization to GAPDH mRNA levels.

### Immunofluorescence staining and flow cytometric analysis

Fluorescence staining was used to identify the expression of CXCR4, Sox2, Nanog, c-Kit, or NKX2.5 after FIR^50 min^ preconditioning. After fixing in 4% PFA at 4°C for 15 min, cells were washed with PBS, permealized with 5% BSA and 0.1% Triton X-100, and incubated with primary antibodies. Fluorescence imaging was performed on the inverted ZEISS Observer.Z1 confocal laser microscope system using 488/405 nm lasers with a 20x objective. All images were selected with sample identities blinded and at least 20 random images were obtained from each well or group.

### Western blot analysis

Ice-cold PRP-PREP protein extraction solution with protease inhibitor cocktail (iNtRON Biotechnology, Inc, Seoul, Korea) was added after FIR treatment of the samples, followed by homogenization using stainless steel beads (Qiagen). An equal amount of protein (50 μg) for each sample was loaded onto a 10 - 12% SDS gel, subjected to electrophoresis, and transferred to the PVDF membranes (Merk Millipore, MA, USA). The membranes were blocked for 2 h at room temperature with 5% nonfat dry milk in PBS containing 0.1% Tween-20, and incubated with primary antibodies (1:1000 and 1:500, respectively) overnight at 4°C. After washing, the membranes were incubated with a horseradish peroxidase-conjugated secondary antibody (1:5000) at RT for 2 h, and then visualized with a chemiluminescence substrate.

### Scratch wound healing assay

BMSCs were seeded in 6-well plates, and the cells were serum starved for 12 h when they had grown to 95% confluence, as previously described^27,28^. A scratch wound was created with a micropipette tip. After FIR^50 min^ preconditioning, cell migration was monitored under a phase contrast microscope (Olympus Optical Co., Tokyo, Japan) using an ocular grid at 0, 8, 16, and 24 h. Cells were then photographed using a DCF300 digital camera (Scopetek, Inc., Hangzhou, China) with ScopePhoto software (Scopetek, Inc.,). The cell migration rate was calculated using the following formula:

$${\rm{Cell}}\,{\rm{migration}}\,{\rm{rate}}=(0\,{\rm{time}}\,{\rm{wound}}\,{\rm{width}}\,-\,{\rm{final}}\,{\rm{wound}}\,{\rm{width}})/10.$$

The migration rate (%) is presented as a percentage, with migration in the BMSCs^con^ set to 100%. All experiments were performed in triplicate and were repeated at least three times.

### Transwell migration assay

To further confirm the increased migration of FIR-preconditioned BMSCs in response to SDF-1α, a cell migration assay was performed using 0.8 μm pore size, 24-well transwell migration chambers coated with Type IV collagen (10 μg/ml) as previously described^27,28^. Briefly, 1 × 10^4^ BMSCs were seeded into the upper transwell chambers containing medium without SDF-1α (20 ng/ml). Then, the chamber was inserted into each well of 24-well plates containing 600 μl basal MSCGM medium supplemented with SDF-1α (20 ng/ml). After FIR^50 min^ preconditioning, the chambers were then incubated for 16 h at 37°C in a 5% CO_2_ incubator. The cells that migrated through to the other side of the membrane were stained with a crystal violet staining solution. Then the absorbance was determined at 590 nm using an ELISA reader (Emax; Molecular Devices, Sunnyvale, CA, USA).

### Statistics

The statistical significance of differences between groups was assessed by analysis of variance (ANOVA) followed by Tukey’s *post hoc tests*. *P* values (*) less than 0.05, *P* values (**) less than 0.01, and *P* values (***) less than 0.001 were considered significant.

## Electronic supplementary material

supplemental information
