# Supplementary material for: Preconditioning with far-infrared irradiation enhances proliferation, cell survival, and migration of rat bone marrow-derived stem cells via CXCR4-ERK pathways
Source: Sci Rep. 2017 Oct 20;7:13718. doi: 10.1038/s41598-017-14219-w (PMC5651919; doi:10.1038/s41598-017-14219-w)
Supplement: Supplementary file 1 — supplemental information [file 41598_2017_14219_MOESM1_ESM.doc]

**Preconditioning with far-infrared irradiation enhances proliferation, cell survival, and migration of rat bone marrow-derived stem cells via CXCR4-ERK pathways**

Yun-Mi Jeong^1^, Xian Wu Cheng^1,2^, Sora Lee^1^, Kyung Hye Lee^1^, Haneul Cho^1^, Jung Hee Kang^1^, Weon Kim^1,*^

^1^Division of Cardiology, Department of Internal Medicine, Kyung Hee University Hospital, Kyung Hee University, Seoul, Republic of Korea

^2^ The Department of Cardiology, Yanbian University Hospital, Yanji, China

**SUPPLEMENTAL INFORMATION**

**Materials**

EZ-Cytox cell viability assay reagent was obtained from DoGenBio Co., Ltd. Hoechst33342, Click-iT®Plus EdU (5-ethynyl-2-deoxyuridine) Alexa Fluor® 488 Flow Cytometry Assay Kit, and BrdU (5-bromo-2-deoxyuridine) were purchased from Thermo Fisher Scientific (Rockford, IL, USA). AccuPower®RocketScript^TM^ Cycle RT PreMix (dN12) and AccuPower®ProFi Taq PCR PreMix was purchased from Bioneer (DaeJeon, Korea). SYBR®Green Mix was obtained from Applied Biosystems (Lincoln, CA, USA). Crystal violet staining solution, H_2_O_2_, PD98059, and AMD3100 octahydrochloride hydrate (CXCR4 inhibitor) were purchased from Sigma (ST. Louis, MO, USA).

Antibodies that recognize Nkx2.5 (ab106923), CXCR4 (ab2074), Nanog (ab106465), goat anti-rabbit IgG H&L (Alexa Fluor®488), and donkey anti-goat IgG H&L (Alexa Fluor®647) were purchased from Abcam (Cambridge, UK). Antibodies specific for BrdU (sc32323) -, c-Kit (sc5535) -, Sox2 (sc20088), and actin (sc47778) were obtained from Santa Cruz Biotechnology, Inc. (Santa Cruz, CA, USA). Anti-p-p44/42 MAPK was obtained from Cell Signaling (Danvers, MA, USA). Antibody specific for c-Kit (p-tyr568/tyr570) was purchased from GeneTex Inc (Isleworth, UK).

**Flow cytometric analysis of BMSCs**

The BMSCs were verified using flow cytometry. After fixing in 4% paraformaldehyde (PFA) solution at 4°C for 15 min, cells were washed three times with PBS supplemented with 5% BSA, 5% FCS, and 0.1% Triton X-100 in PBS (buffer A), and then incubated with primary antibodies against CD105, CD166, CD31, and CD45 at 4°C overnight. Flow cytometry was performed using the FACSCalibur flow cytometer (Becton Dickinson, USA) and CellQuest software.

**Cell proliferation analysis**

Cell proliferation assays were performed with EZ-Cytox cell viability assay kit (DoGEN, Seoul, Korea), EdU, and Brd U assay. For EZ-Cytox assays, BMSCs were subjected (or not) to FIR at the indicated irradiation parameters, and grown for 1, 2, 3, or 4 days. After the indicated times, the culture medium was removed, and replaced with EZ-Cytox solution with fresh medium for 2 h at 37°C in 5% CO_2_ incubator. Absorbance was determined at 490 nm using an ELISA reader (Emax; Molecular Devices, Sunnyvale, CA, USA). The assay was repeated three times. To counter the cell numbers of BMSCs^con^ and BMSCs^FIR 50 min^ with or without SDF-1α at the indicated time points, cell proliferation was monitored under a phase contrast microscope (Olympus Optical Co., Tokyo, Japan). Cells were then photographed using a DCF300 digital camera (Scopetek, Inc., Hangzhou, China) with ScopePhoto software (Scopetek, Inc.,). All images were selected with sample identities blinded and at least 20 random images were obtained from each well or group.

For BrdU-cell labeling in BMSCs^con^ and BMSCs^FIR 50 min^, glass-bottom-culture-dishes were used for the BrdU^+^ proliferating cells for three days. The cells were labeled in culture with 10 μM BrdU for 24 h prior to collection. The cells were fixed in 4% PFA, washed with PBS three times for 5 min with shaking, blocked with 3% BSA in PBS for 1 h, and incubated with anti-Brd U AlexaFluor®488 monoclonal antibody (1:100) in 0.5% BSA-PBST overnight at 4°C. Fluorescence imaging was performed on an inverted ZEISS Observer.Z1 confocal laser microscope system using 488/405 nm lasers with a 20x objective.

For the EdU-based S-phase assay, BMSC proliferation was evaluated with the Click-iT® Plus EdU Alexa Fluor ® 488 Flow Cytometry Assay Kit (Molecular Probes by Life Technologies) according to the manufacturer’s instructions. Briefly, BMSCs ^con^ and BMSCs ^FIR 50 min^ were incubated for the indicated times, and then treated with EdU (10 μM). The cells were collected after 24 h of culture at 37°C in 5% CO_2_. After fixation with 1% paraformaldehyde (PFA) at 4°C for 15 min, the cells were washed three times with PBS, permeabilized with Click-iT®saponin-based permeabilization reagent, and incubated with the Alexa Fluor 488 EdU solution for 30 min at RT. The relative fluorescence intensity of EdU^+^cells was acquired using a FACSCalibur flow cytometer (Becton Dickinson, USA) and CellQuest software.

**Oxidative stress and TUNEL analysis**

BMSCs were pretreated with FIR for 50 min. After 2 h, the BMSC medium was replaced with serum-free medium supplemented with H_2_O_2_ (600 μM) for another 24 h. The cell viability was assessed with the EZ-Cytox assay. To further confirm the protective effects of FIR, apoptotic cells in BMSCs^con^ and BMSCs^FIR 50 min^ were evaluated after 24 h by terminal deoxynucleotidyl transferase-mediated dUTP-biotin nick end labeling (TUNEL) assay using the Apo-Direct TUNEL assay kit (Millipore), as previous described (Darzynkiewicz et al). Cells were analyzed by a FACSCalibur flow cytometer (Becton Dickinson, USA) and CellQuest software.

**LEGENDS FOR SUPPLEMENTAL INFORMATION**

**
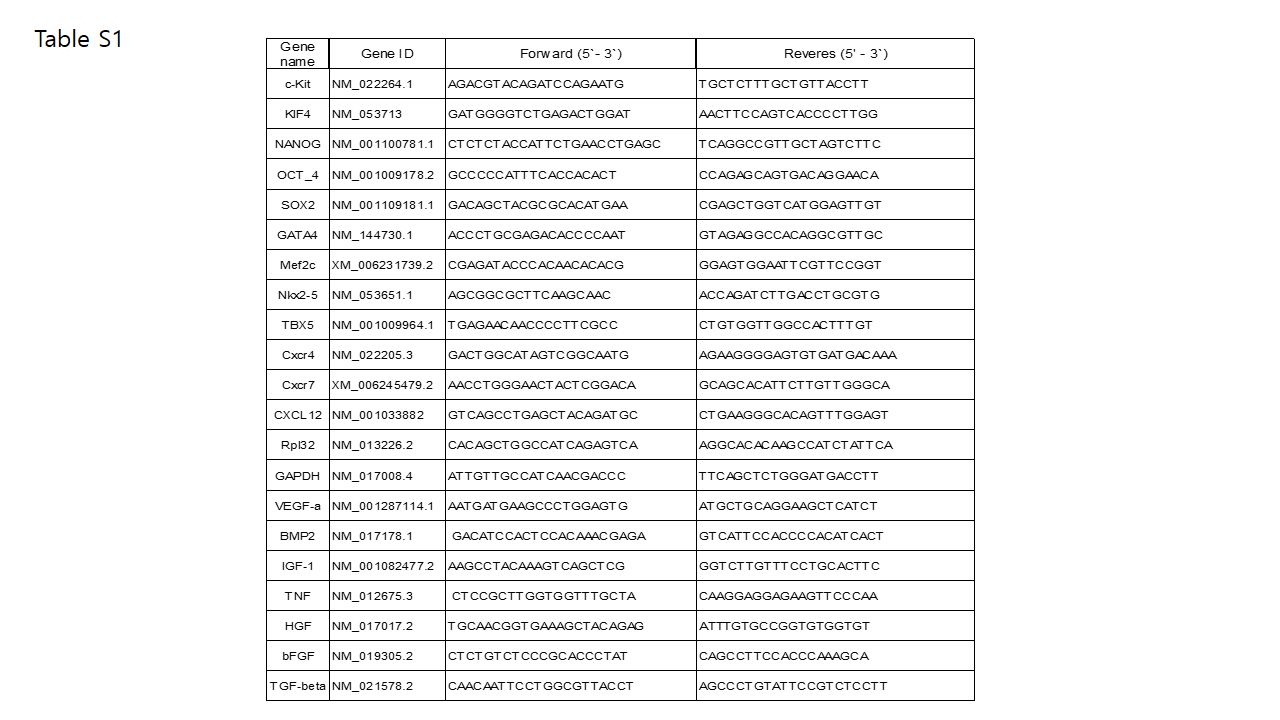
**

**Table S1. Primers**


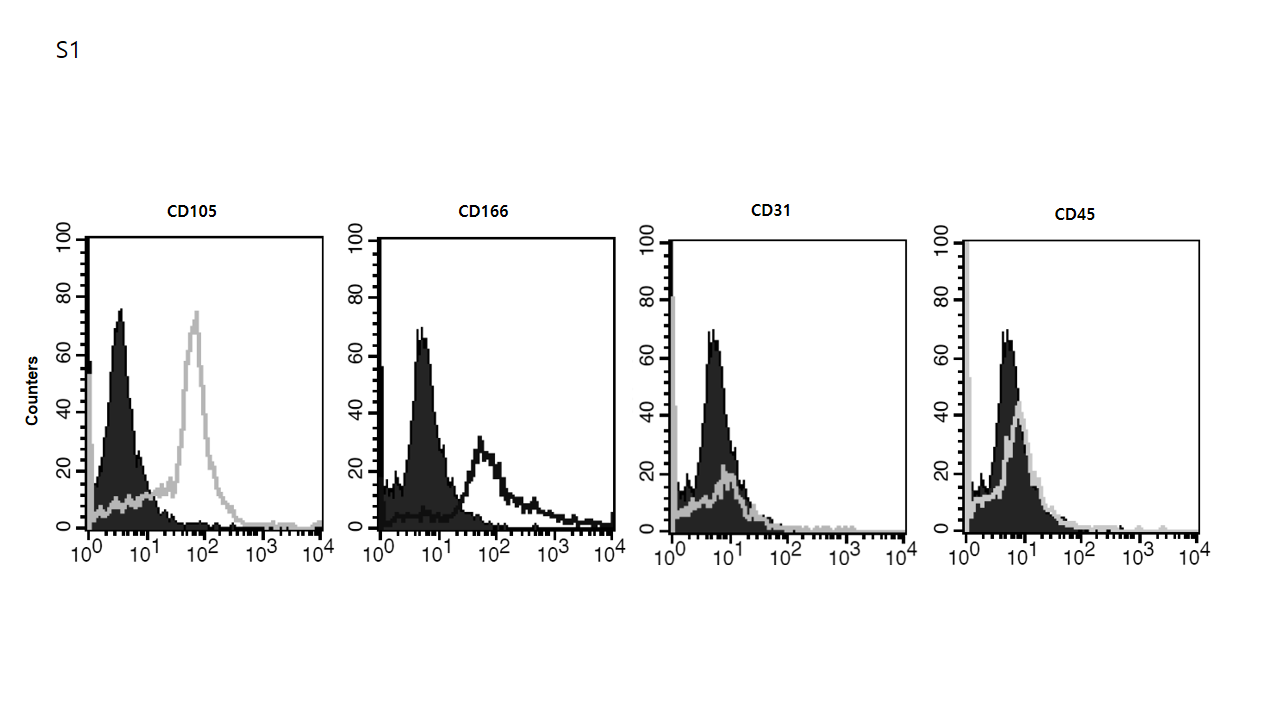


**Figure S1. Characterization of BMSCs.** Representative images of cell surface marker expression. Isolated rat BMSCs were identified by FACS analysis with their markers (CD105^+^/CD166^+^/CD45^-^/CD31^-^). As shown in Fig. S1, BMSCs positively expressed CD105 and CD166, but did not express the endothelial marker CD31 or the hematopoietic lineage marker CD45. Three independent experiments were performed.


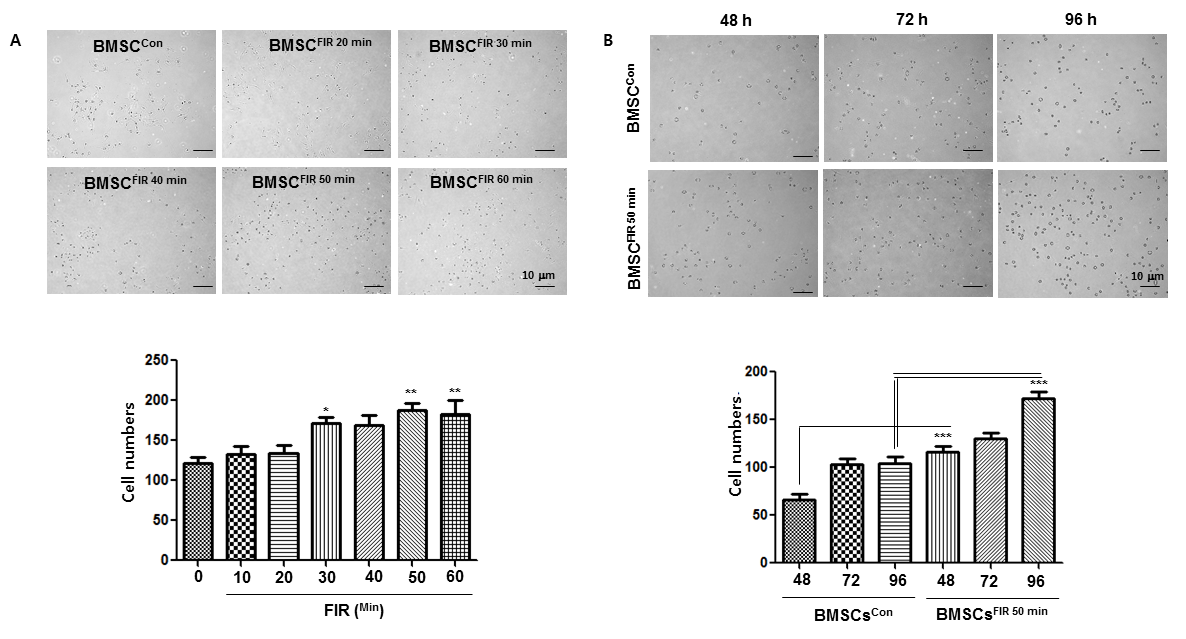


Figure S2. FIR significantly stimulates BMSC proliferation in a dose/time dependent. (A) Images of BMSCs^con^ and BMSCs^FIR50 min^ in a dose-dependent manner. Graph of proliferation rates, using a cell counter at the indicated dose points for representative samples, shown as percentage increases compared to BMSCs^con^ at t = 72 h. (B) Time lapse images of BMSCs^con^ and BMSCs^FIR50 min^. Graph of proliferation rates, using a cell counter at the indicated time points for representative samples, shown as percentage increases compared to BMSCs^con^ at t = 48 h.


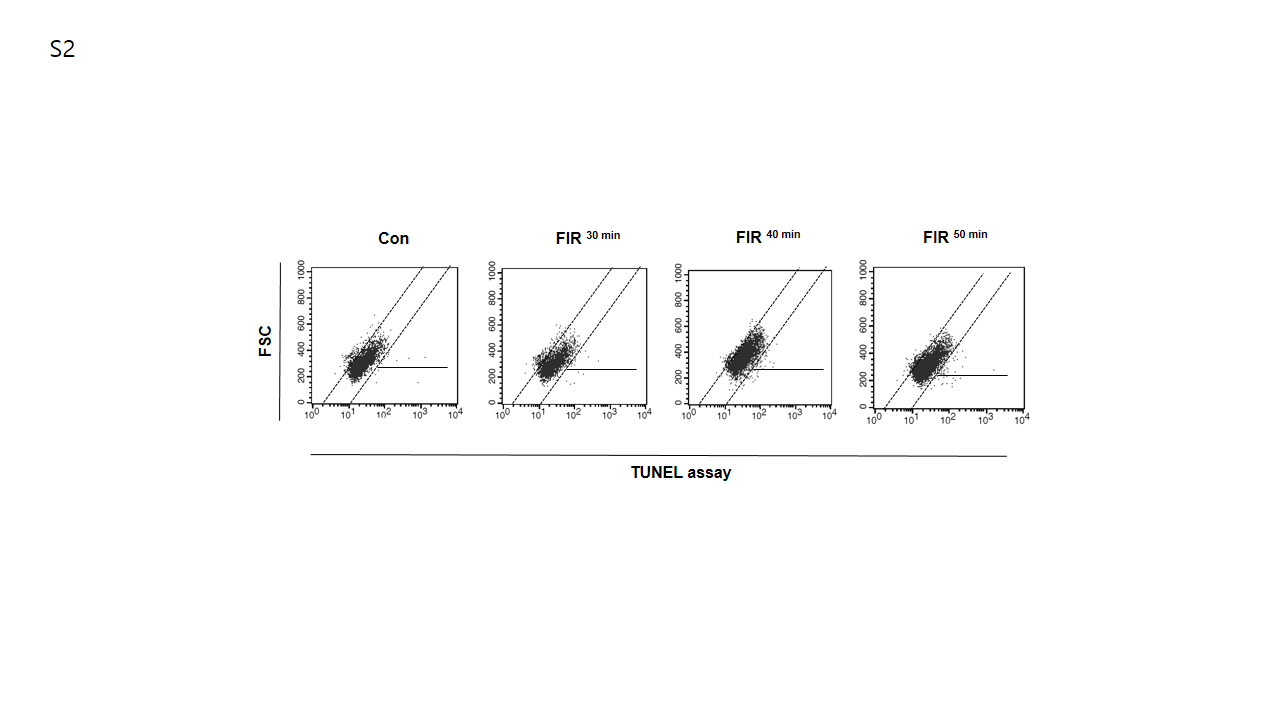


**Figure S3. The effect of FIR on BMSC viability.** (A) After FIR for 30, 40, or 50 min, BMSC viability was measured by the TUNEL assay. FIR was not cytotoxic to BMSCs.


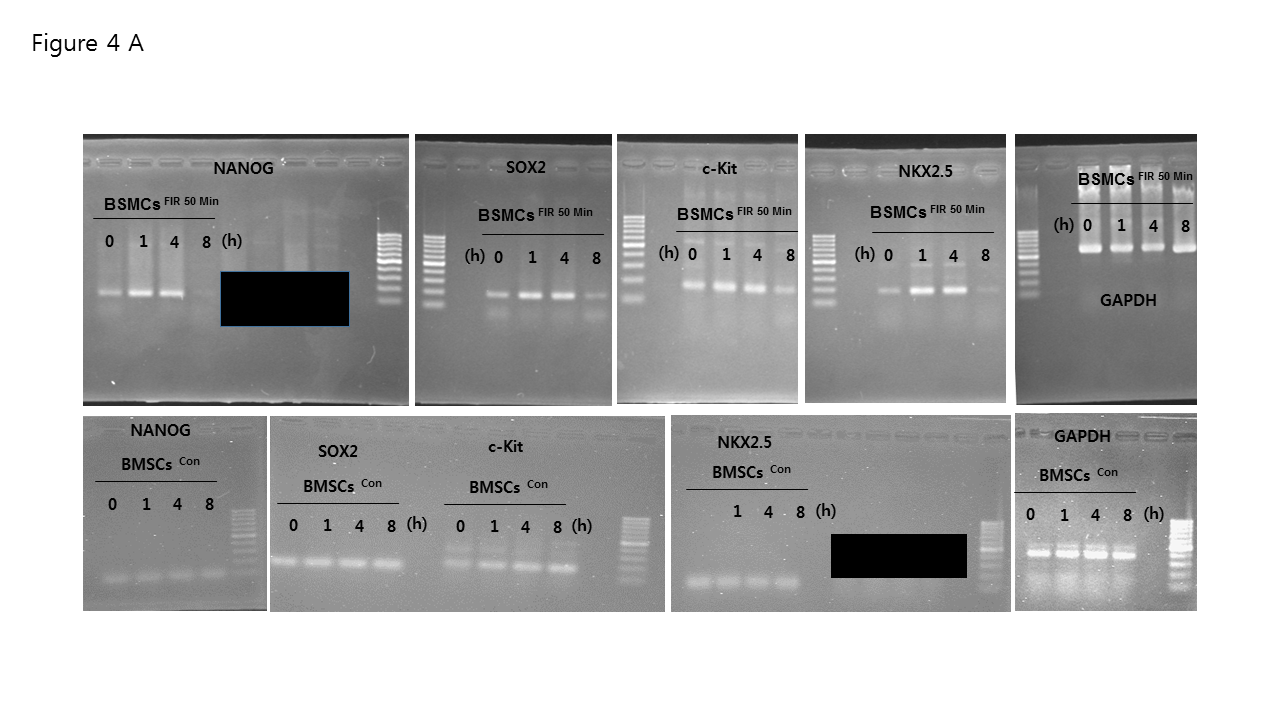


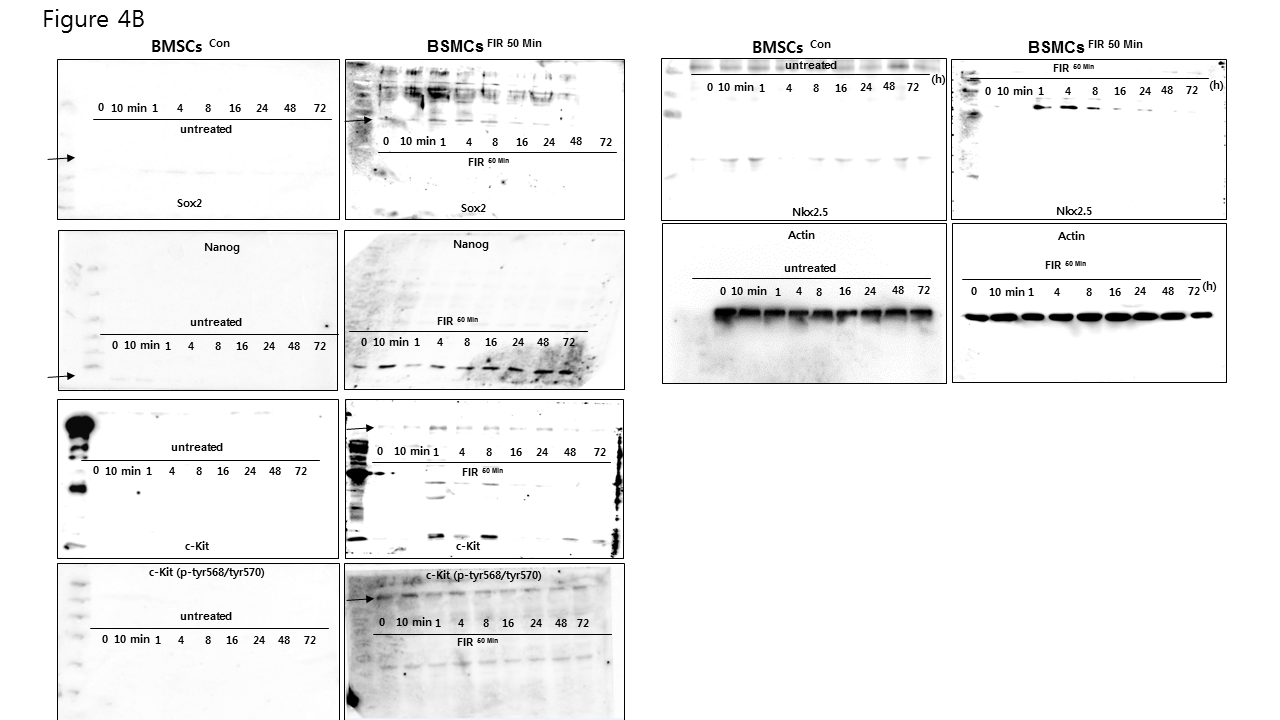


**Full-length gels/blots images of main Figure 4A and 4B.** (Figure 4A) Full-length gel images. (Figure 4B) Full-length Western blot images.


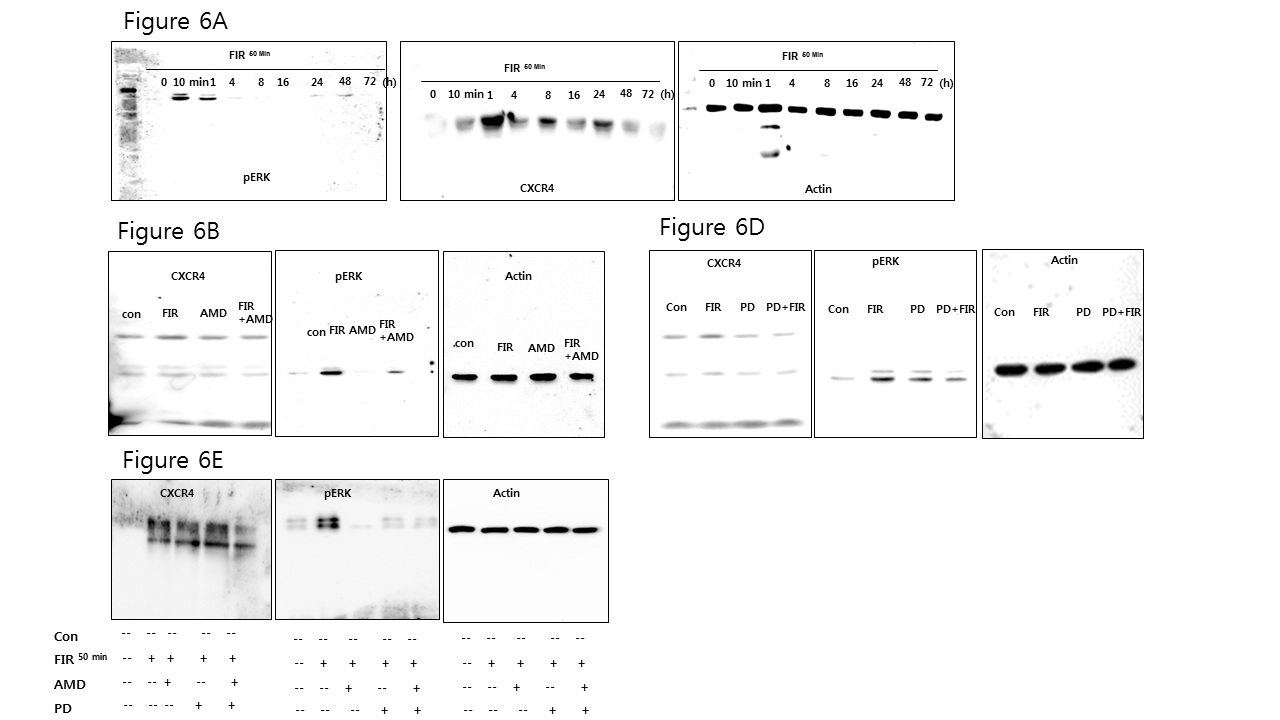


**Full-length Western blots images of main Figure 6A – 6C.**
